# Crystal structure, Hirshfeld surface analysis and DFT study of 2-(2-isopropyl-5-methyl­cyclo­hex­yloxy)-2-oxoethyl benzoate

**DOI:** 10.1107/S2056989026007528

**Published:** 2026-07-23

**Authors:** Zulfiya Khalmuratova, Ugilay Abdurakhmanova, Alimjan Matchanov, Odil Choriyev, Batirbay Torambetov, Alisher Eshimbetov, Jamshid Ashurov

**Affiliations:** a Karakalpak Scientific Research Institute of Natural Sciences, Karakalpak Branch of the Academy of Sciences of the Republic of Uzbekistan, 41 Berdakh Avenue, Nukus 230100, Republic of Karakalpakstan, Uzbekistan; b Gulistan State University, 4th Microdistrict, Gulistan, Syrdarya Region 120100, Uzbekistan; c Institute of Bioorganic Chemistry, Academy of Sciences of Uzbekistan, 100125, M. Ulugbek Str 83, Tashkent, Uzbekistan; d National University of Uzbekistan named after Mirzo Ulugbek 4 University St Tashkent 100174 Uzbekistan; e Termez University of Economics and Service, 4-b Farovon Street, Termez 190111, Uzbekistan; Vienna University of Technology, Austria

**Keywords:** menthol derivative, crystal structure, electrostatic potential, frontier orbitals, C—H⋯O inter­action, weak inter­molecular inter­actions

## Abstract

The title menthol-derived ester, C_19_H_26_O_4_, was synthesized by chloro­acetyl­ation of menthol followed by reaction with sodium benzoate. It crystallizes in the Sohncke space group *P*2_1_ with one mol­ecule in the asymmetric unit. The cyclo­hexane ring adopts a chair conformation, and the benzoate fragment is essentially planar. The oxoethyl bridge shows a *gauche* conformation about the O—CH_2_ bond and an *anti* conformation about the CH_2_—C bond. In the crystal, the packing is mainly consolidated by a weak C—H⋯O contact linking mol­ecules into chains parallel to [100]. Hirshfeld surface analysis indicates that H⋯H contacts make the largest contribution to the crystal cohesion, followed by O⋯H/H⋯O and C⋯H/H⋯C contacts, consistent with the hydro­phobic menthyl entity. DFT calculations show reasonable agreement between the refined and optimized structures. Frontier-orbital and electrostatic-potential analyses identify the benzoate ester and carbonyl oxygen atoms as the main electronically active regions.

## Abstract 2

The packing in the crystal structure of the title compound is mainly consolidated by a weak C—H⋯O contact linking mol­ecules into chains parallel to [100] and van der Waals inter­actions.

## 1. Chemical context

Menthol, (1*R*,2*S*,5*R*)-2-isopropyl-5-methyl­cyclo­hexan-1-ol, is a naturally occurring cyclic monoterpene alcohol and one of the major constituents of peppermint and cornmint essential oils obtained from *mentha piperita L*. and *mentha arvensis L*. Because of its characteristic cooling sensation, pleasant mint-like odour and favourable safety profile, menthol is widely used in the pharmaceutical, cosmetic, food and flavouring industries. Its cooling and refreshing effect is mainly associated with activation of cold-sensitive thermoreceptors, particularly the TRPM8 ion channel, which also contributes to the analgesic action of menthol-containing topical preparations (Kamatou *et al.*, 2013 ▸; Farco & Grundmann, 2013 ▸; Pergolizzi *et al.*, 2018 ▸; Li *et al.*, 2022 ▸). Kamatou *et al.* (2013 ▸) described menthol as a simple monoterpene with remarkable biological properties and emphasized its importance as a natural compound with broad industrial and medicinal relevance.

In addition to its sensory properties, menthol has attracted considerable attention because of its anti­microbial, anti­oxidant, anti-inflammatory, analgesic and anti­cancer-related activities. Recent reviews have compiled the biochemical, pharmacological and clinical aspects of peppermint and menthol, including their therapeutic potential and safety considerations (Kazemi *et al.*, 2025 ▸; Başer *et al.*, 2025 ▸).

Menthol is not only an important flavouring and cooling agent, but also a biologically relevant mol­ecular scaffold for the design of new derivatives with modified physicochemical and pharmacological properties. Chemical modification of the hydroxyl group of menthol is a common and well-established strategy for obtaining menthyl ester derivatives with altered lipophilicity, conformational behaviour, electrostatic properties and biological activity. Menthyl esters are important as fragrance and flavour compounds, chiral auxiliaries, prodrug-like fragments, penetration-enhancing agents and stereochemically defined substituents in organic synthesis. Mansurov *et al.* (2025 ▸) recently investigated (–)-menthol and a series of menthyl esters, including menthyl acetate, propionate, butyrate, valerate and hexa­noate, using quantum-chemical calculations, mol­ecular docking and related computational approaches. They showed that variation of the ester side chain influences electrostatic properties, lipophilicity and predicted binding behaviour toward biologically relevant protein targets.

The biological relevance of menthol esterification is also supported by earlier experimental studies. Samarasekera *et al.* (2008 ▸) synthesized several menthol derivatives, including menthyl chloro­acetate, menthyl di­chloro­acetate and menthyl cinnamate, and evaluated the insecticidal activity against mosquito species such as *culex quinquefasciatus*, *aedes aegypti* and *anopheles tessellatus*. These results showed that esterification of menthol can significantly influence biological activity and may provide derivatives with useful bioactivity profiles.
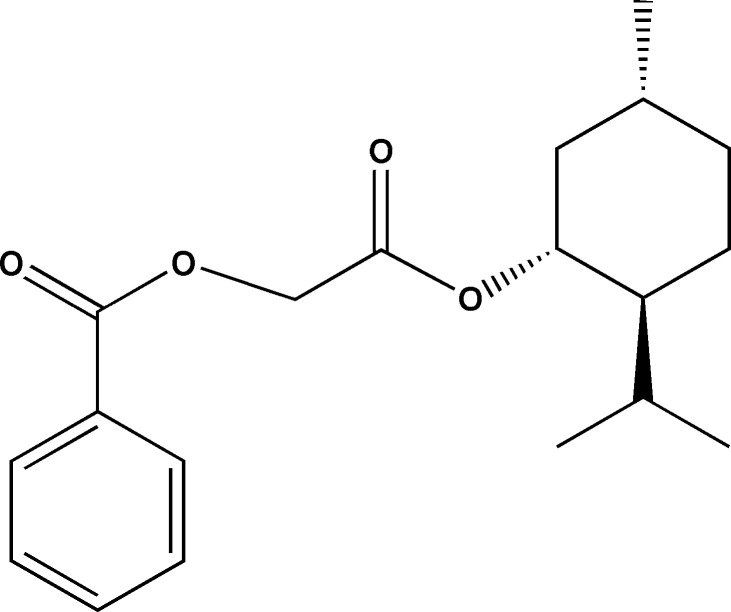


In the context given above, the title compound, 2-(2-isopropyl-5-methyl­cyclo­hex­yloxy)-2-oxoethyl benzoate, was synthesized and its mol­ecular and crystal structure determined by single-crystal X-ray diffraction, complemented by Hirshfeld surface analysis and DFT calculations.

## 2. Structural commentary

**Figure 1:**
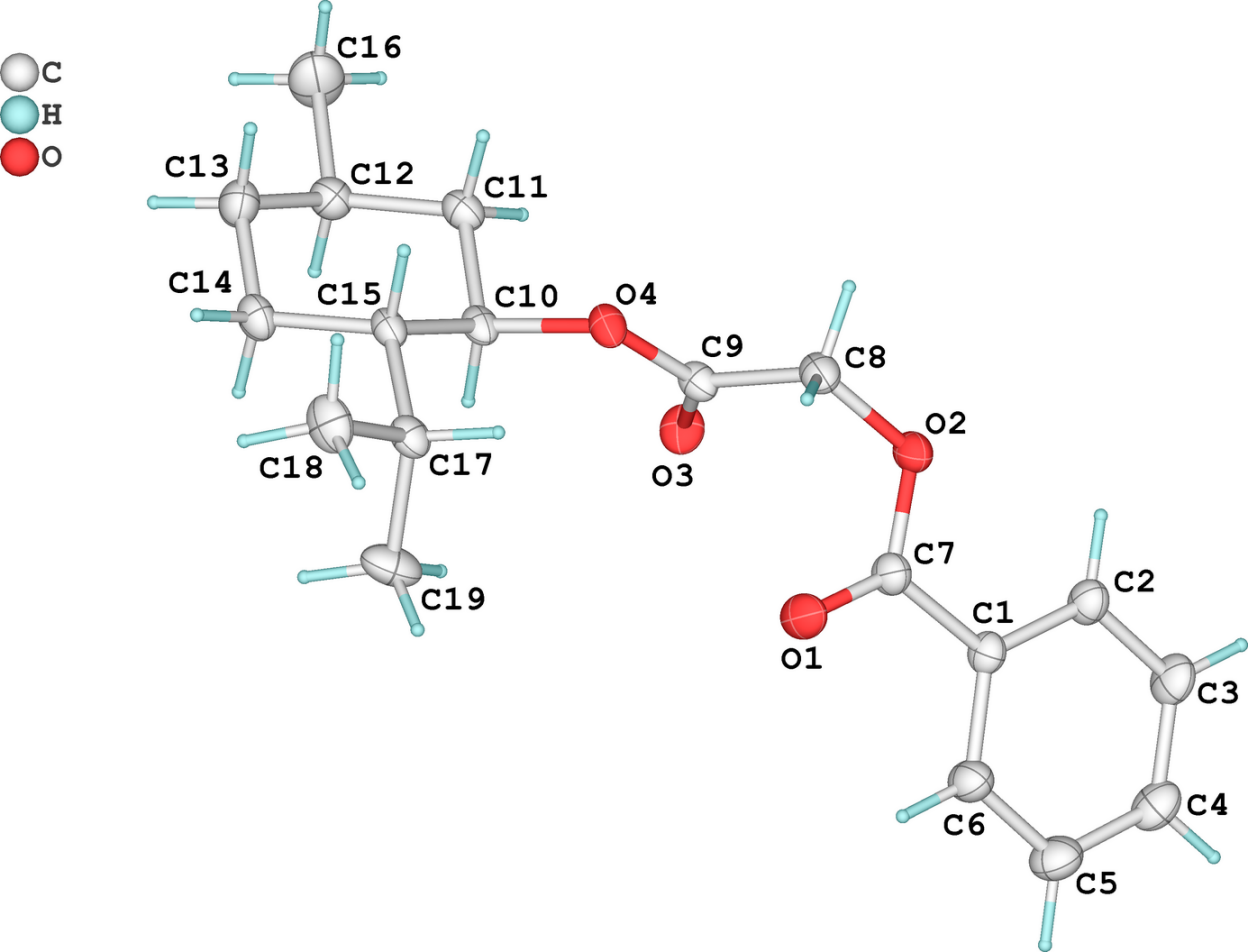
The mol­ecular structure of the title compound, showing the atom-labelling scheme. Displacement ellipsoids for non-H atoms are drawn at the 30% probability level.

The title compound, C_19_H_26_O_4_, crystallizes as a neutral mol­ecule in the monoclinic Sohncke space group *P*2_1_, with one mol­ecule in the asymmetric unit. It is a new menthol-derived ester in which the menthyl cyclo­hexyl fragment is linked to a terminal benzoate group through an ester–methyl­ene bridge. The mol­ecule combines a bulky hydro­phobic cyclo­hexyl moiety, a flexible oxoethyl linker and an aromatic benzoate fragment, features that may influence its conformation and weak inter­molecular inter­actions in the solid state. The mol­ecular structure of the title compound is shown in Fig. 1 ▸.

The non-centrosymmetric space group is consistent with the chiral menthyl moiety. The mol­ecule consists of three main fragments: a benzoyl group [Ph—C(=O)–], a central oxo­acetate linker [–OCH_2_C(=O)–], and a menth­yloxy moiety incorporating the chiral cyclo­hexane ring. The two ester groups are clearly defined by the bond lengths. In the benzoyl ester fragment, the carbonyl bond O1=C7 is 1.204 (7) Å, whereas the ester bond C7—O2 is longer at 1.325 (8) Å. In the second ester group, the O3=C9 carbonyl bond is 1.177 (6) Å and the C9—O4 single bond is 1.347 (7) Å. These values are typical for ester functionalities and indicate normal localization of C=O and C—O bonds. The phenyl ring, C1–C6, is essentially planar, with aromatic C—C distances in the range 1.355 (13) to 1.390 (13) Å. The benzoyl carbonyl group is almost co-planar with the aromatic ring, as shown by the torsion angles C2—C1—C7—O1 of −177.2 (6)° and C6—C1—C7—O2 of −176.4 (6)°. This arrangement favours conjugation between the phenyl ring and the carbonyl group. The oxoethyl bridge adopts a *gauche* conformation between the two ester fragments. This is indicated by the torsion angle C7—O2—C8—C9 of −69.7 (7)°. In contrast, the second ester fragment is nearly planar around the carbonyl group, with C10—O4—C9—O3 of −0.1 (8)° and C10—O4—C9—C8 of −179.5 (5)°. The cyclo­hexyl ring of the menthyl fragment, formed by atoms C10–C15, adopts a chair conformation. The C—C bond lengths within the ring fall in the normal range from 1.498 (10) to 1.533 (7) Å. The alternating ring torsion angles, from about −57 to +58°, are characteristic of a chair cyclo­hexane ring. The Cremer–Pople puckering parameters *Q* = 0.569 (7) Å and *θ* = 0.9 (7)° indicate an almost ideal chair conformation. The menthyl fragment contains three stereogenic centres at C10, C12 and C15. The crystallographic model corresponds to the expected stereochemical arrangement for a menthol-derived entity.

Overall, the bond lengths and angles in the mol­ecular structure confirm the formation of a menthol-derived diester containing a planar benzoyl fragment, two ester carbonyl groups and a cyclo­hexyl ring in a nearly ideal chair conformation.

## 3. Supra­molecular features

**Figure 2:**
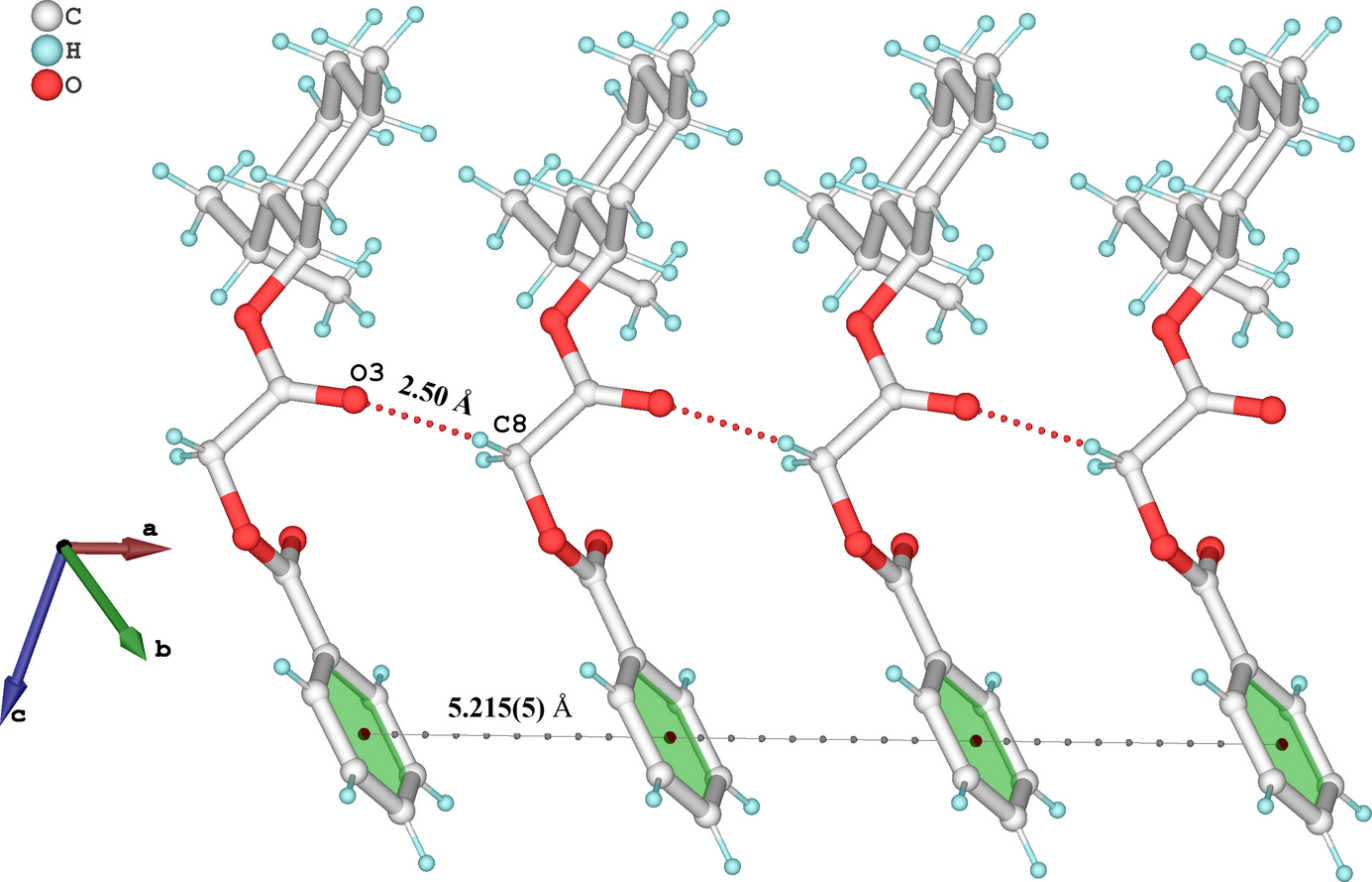
A partial view of the crystal packing of the title compound, showing C—H⋯O hydrogen bonds and weak long-range π–π contacts.

**Figure 3:**
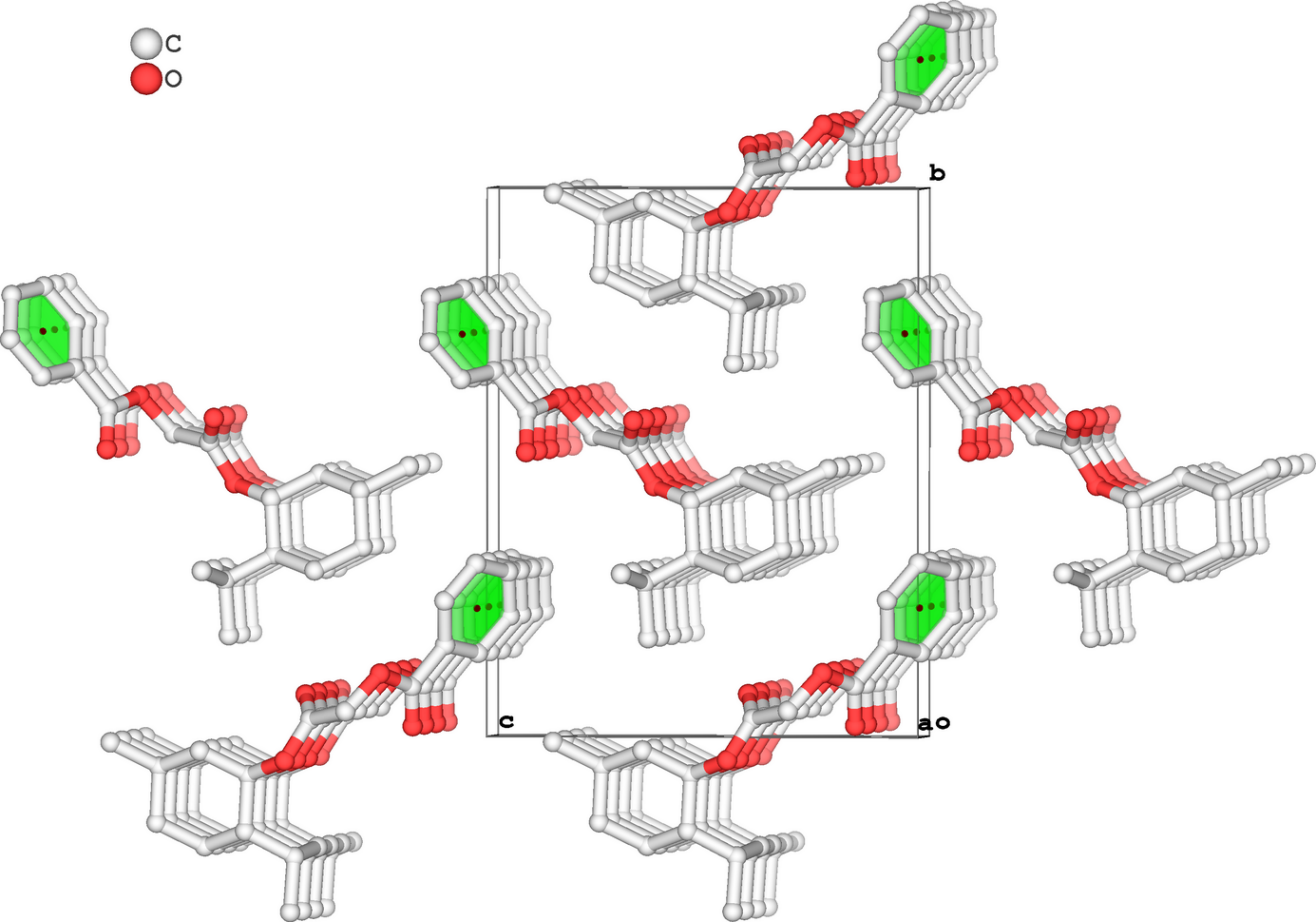
Crystal packing of the title compound viewed approximately along [100], with H atoms omitted for clarity.

The mol­ecule does not contain donor groups for classical hydrogen-bonding. The crystal packing is consolidated mainly by a weak C—H⋯O contact involving the methyl­ene group and the carbonyl oxygen atom of a neighbouring mol­ecule (Table 1 ▸) and van der Waals inter­actions, as illustrated in Fig. 2 ▸. An alternative view of the crystal packing is shown in Fig. 3 ▸. An additional intra­molecular C17—H17⋯O4 contact is observed, with H⋯O = 2.48 Å, C⋯O = 2.889 (7) Å and an angle of 105°. Owing to this small angle, this contact should be regarded as weak, although it may help to fix the relative orientation of the isopropyl and ester moieties. The aromatic rings do not participate in significant π–π stacking inter­actions. Although neighbouring phenyl rings are almost parallel, the centroid-to-centroid separations are long [centroid–centroid distance = 5.215 (5) Å] and the slippage is large, 4.11 Å, preventing effective overlap of the aromatic rings. Thus, π–π inter­actions make no meaningful contribution to the packing. Overall, the packing is governed by weak C—H⋯O contacts, van der Waals inter­actions and shape complementarity between the hydro­phobic phenyl and menthyl fragments. The ester carbonyl oxygen atoms act as weak acceptor sites, while aliphatic and aromatic C—H groups provide weak donor contacts.

## 4. Hirshfeld surface analysis

Hirshfeld surface analysis was carried out with *CrystalExplorer* (Spackman *et al.*, 2021 ▸) at high resolution. The calculated Hirshfeld surface has a volume of 457.47 Å^3^ and an area of 392.81 Å^2^. The globularity value of 0.731 and asphericity value of 0.252 indicate that the mol­ecular surface is far from spherical, reflecting the elongated benzoate ester group and bulky hydro­phobic menthyl fragment.

**Figure 4:**
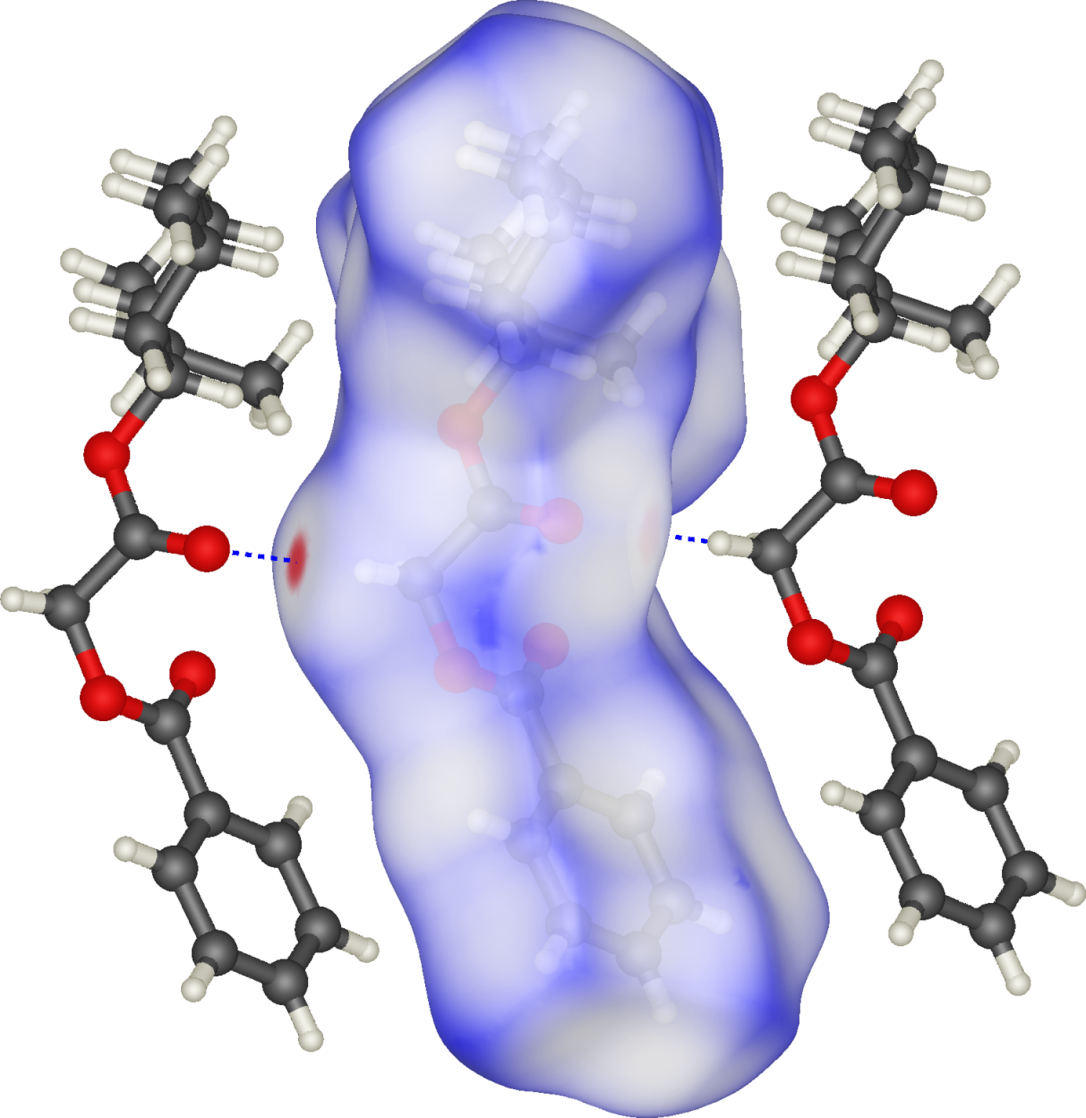
Hirshfeld surface of the title compound mapped over *d*_norm_, showing the inter­molecular C—H⋯O contacts.

The *d*_norm_ surface (Fig. 4 ▸) shows only limited negative regions. This agrees with the absence of strong classical hydrogen-bonding features. The red regions on the *d*_norm_ surface are expected mainly near the carbonyl oxygen atoms and neighbouring C—H groups, corresponding to weak C—H⋯O contacts. The shape-index surface does not provide evidence for efficient π–π stacking, in line with the long centroid–centroid separation and the absence of a significant C⋯C fingerprint contribution.

**Figure 5:**
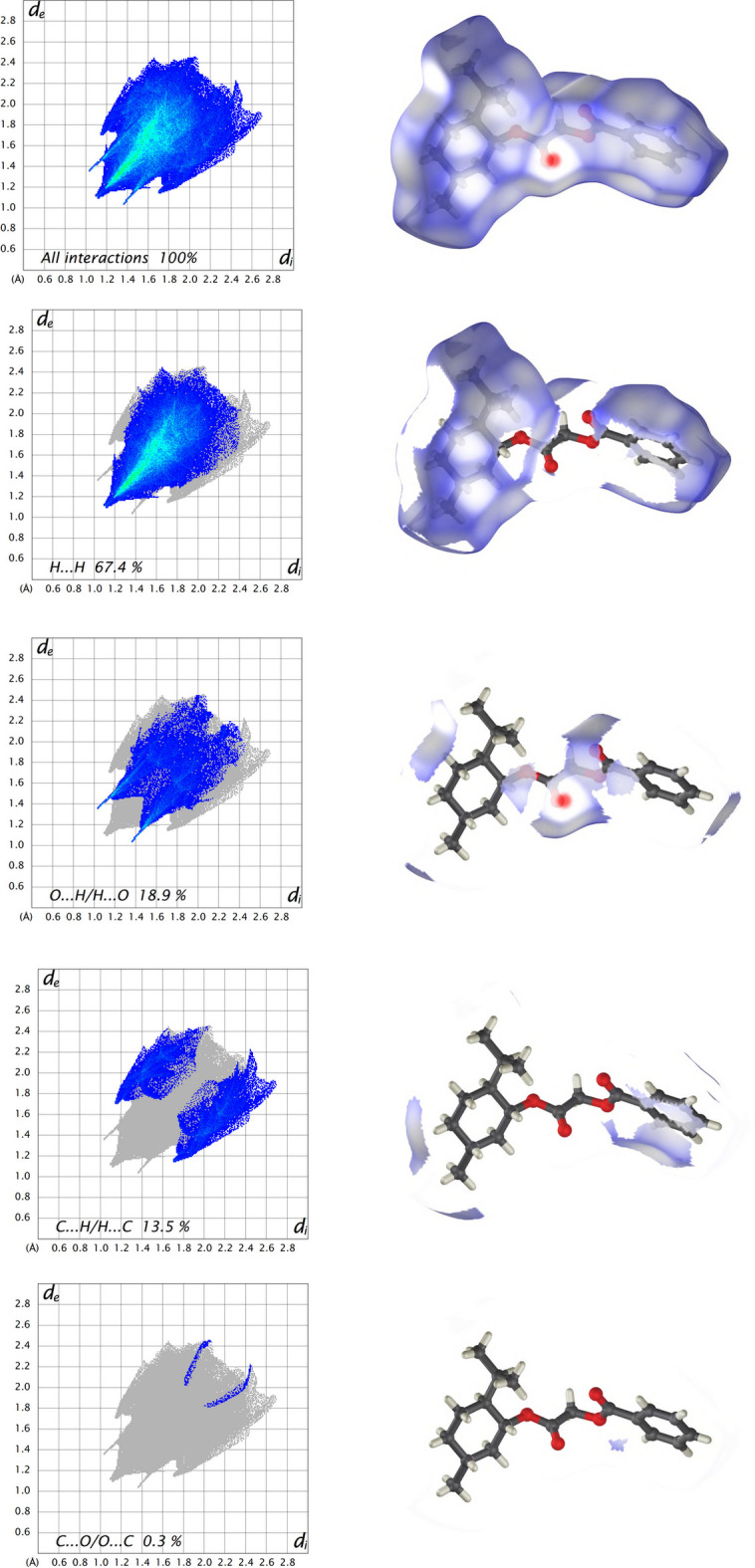
Hirshfeld surface mapped over *d*_norm_ and the corresponding two-dimensional fingerprint plots for the title compound. The relative contributions of H⋯H, O⋯H/H⋯O, C⋯H/H⋯C and C⋯O/O⋯C contacts to the Hirshfeld surface are indicated

The two-dimensional fingerprint plots (Fig. 5 ▸) show that H⋯H contacts dominate the packing, contributing 67.4% of the Hirshfeld surface. This large value is expected for a mol­ecule rich in aliphatic hydrogen atoms. The next largest contribution is from O⋯H/H⋯O contacts, 18.9%, which arise from weak C—H⋯O inter­actions involving the ester oxygen atoms. C⋯H/H⋯C contacts contribute with 13.5%, indicating weak contacts between the aromatic/aliphatic carbon framework and hydrogen atoms. The C⋯O/O⋯C contribution is very small, 0.3%, and C⋯C contacts are negligible. Thus, the crystal packing is governed predominantly by van der Waals H⋯H contacts, with a smaller but structurally relevant contribution from weak C—H⋯O inter­actions.

## 5. DFT calculations

Density functional theory (DFT) calculations were carried out in order to compare the experimentally determined mol­ecular structure with the gas-phase optimized structure and to evaluate its frontier mol­ecular orbitals and electrostatic potential distribution. The calculations provide quantum-chemical support for the structural analysis and help identify the mol­ecular fragments that are most relevant to inter­molecular inter­actions in the crystal.

**Figure 6:**
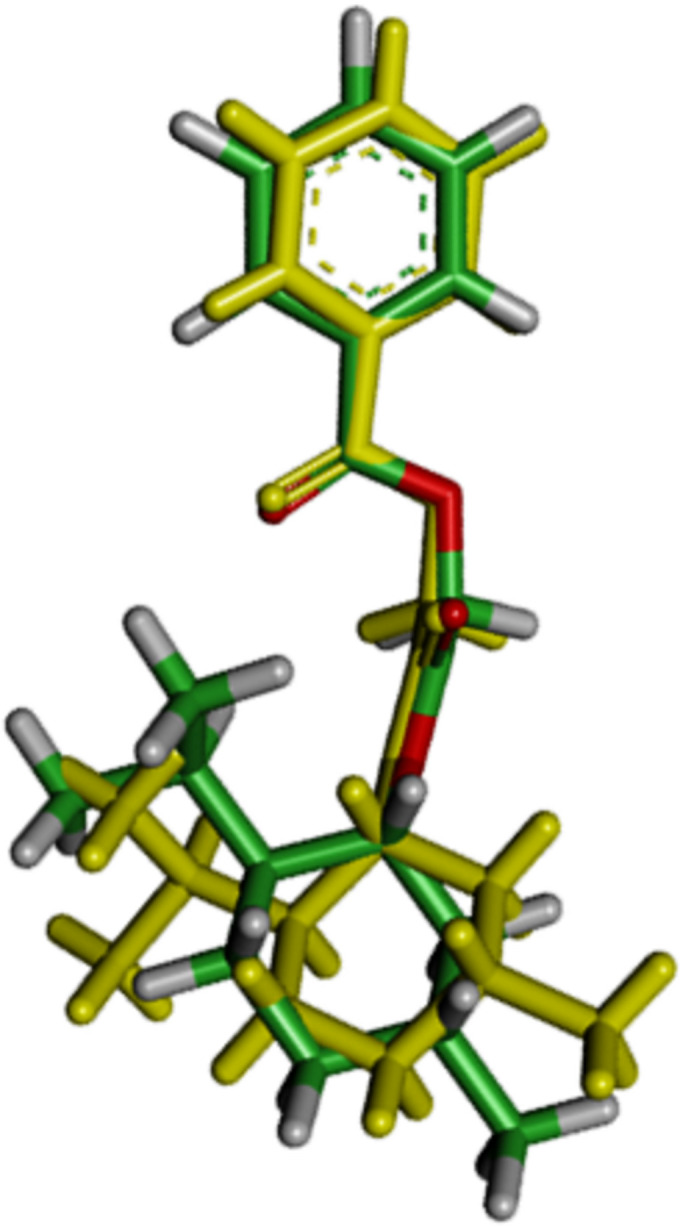
Overlay plot of the refined (yellow) and DFT-optimized (green) structures; oxygen atoms are shown in red for the latter.

The mol­ecular structure obtained from the crystal structure refinement was used as the initial model for geometry optimization performed in the gas phase at the B3LYP/def2-TZVP level of theory with Grimme D3BJ dispersion correction (Grimme *et al.*, 2011 ▸) using the *ORCA* software package (Neese, 2022 ▸). The input file was prepared using *Avogadro* (Hanwell *et al.*, 2012 ▸). An overlay plot of the experimentally determined structure (XRD) with the DFT-optimized structure is shown in Fig. 6 ▸.

The superposition of the XRD and DFT structures gives a root-mean-square-deviation of 0.97 Å, indicating reasonable agreement between the solid-state and optimized gas-phase structures. The main conformational difference is associated with rotation of the cyclo­hexyl fragment about the ester C—O bond. In the optimized structure, the corresponding dihedral angle is 111.5°, whereas the refined structure shows a value of 149.3 (5)°, corresponding to an approximate rotation of 38°. This difference is expected because the experimental mol­ecular conformation is stabilized by inter­molecular inter­actions and crystal-packing effects, whereas gas-phase optimization allows the mol­ecule to adopt an isolated low-energy conformation.

**Figure 7:**
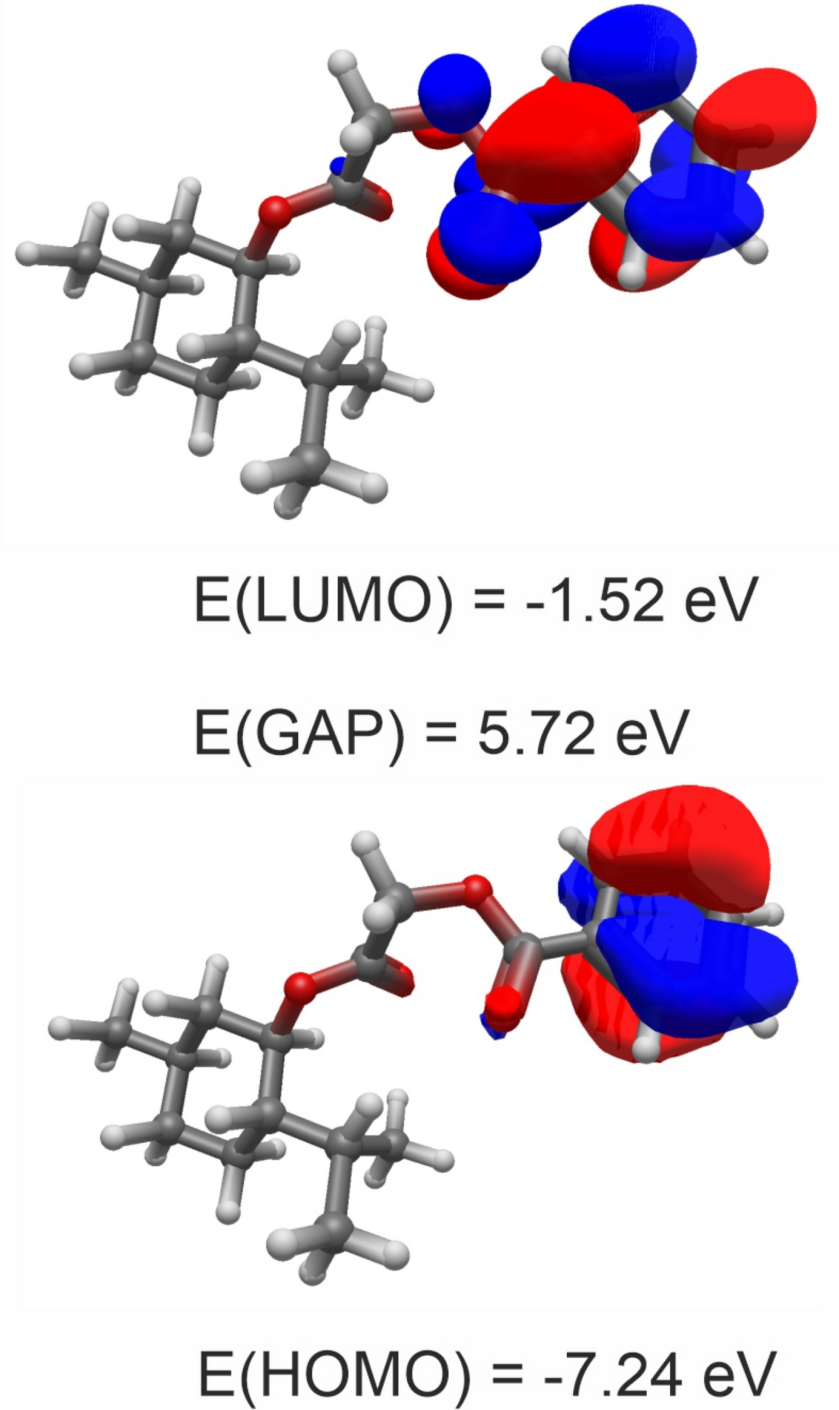
Electron-density distributions of the frontier mol­ecular orbitals of the title compound, calculated at the DFT/def2-TZVP level

The calculated frontier mol­ecular orbitals (FMOs) are shown in Fig. 7 ▸. The highest occupied mol­ecular orbital (HOMO) energy is −7.24 eV, whereas the lowest unoccupied mol­ecular orbital (LUMO) energy is −1.52 eV. The resulting HOMO–LUMO energy gap is 5.72 eV. This gap indicates a relatively stable electronic structure, where the presence of the benzoate fragment lowers the LUMO level and increases the contribution of the aromatic ester unit to the frontier orbital distribution.

The HOMO and LUMO electron densities are mainly localized on the benzoate fragment, including the aromatic ring and adjacent carbonyl-containing ester group. This localization shows that the aromatic acyl fragment is the principal electronically active part of the mol­ecule. Therefore, weak inter­molecular contacts involving the benzoate ring and carbonyl oxygen atoms can be expected to play an important role in the crystal packing.

**Figure 8:**
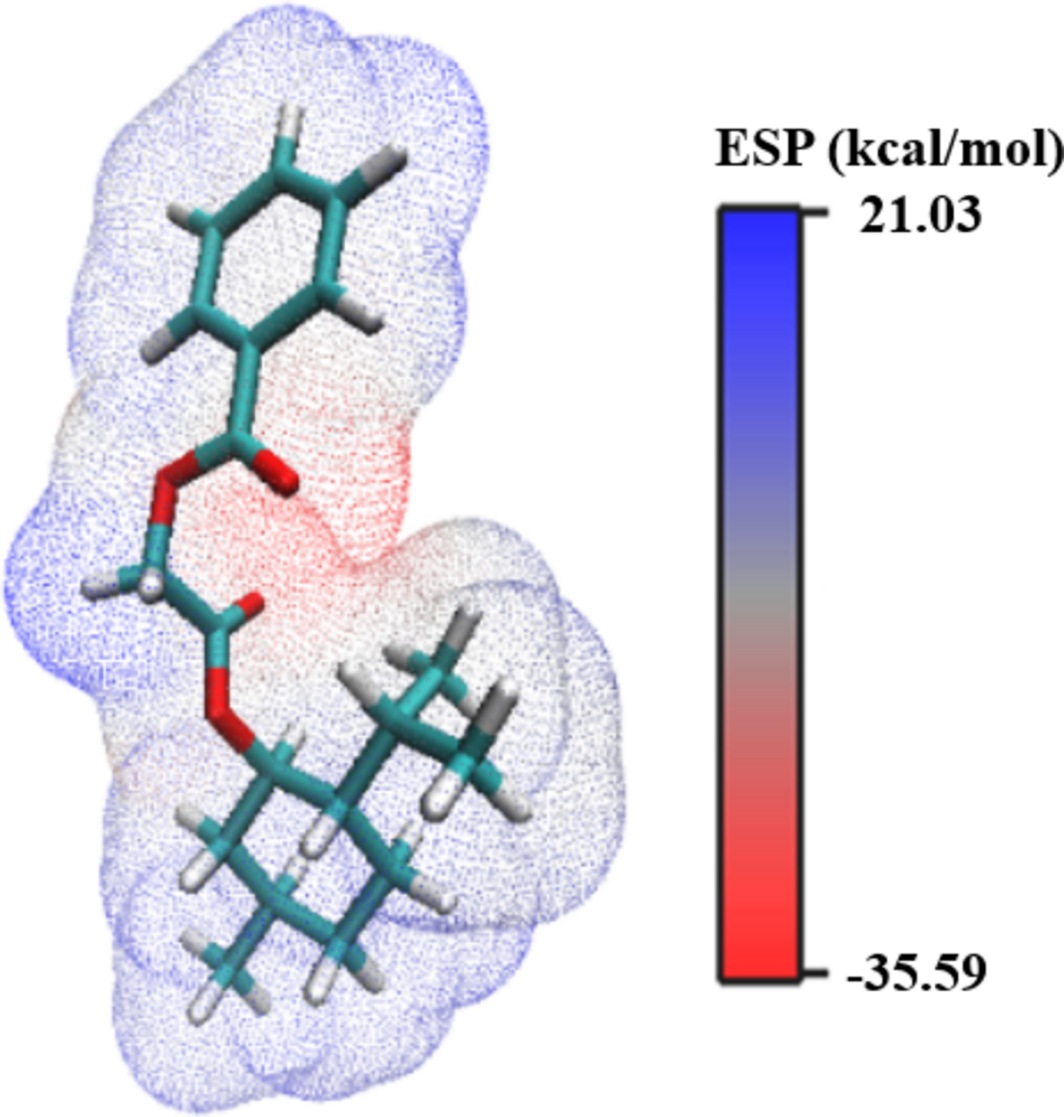
Electrostatic potential (ESP) mapped onto the electron-density surface of the title compound. Colours indicate the electrostatic potential from red (minimum) to blue (maximum), in kcal mol^−1^.

The electrostatic potential (ESP) surface was analysed to locate the electron-rich and electron-deficient regions of the mol­ecule (Murray & Politzer, 2011 ▸). As shown in Fig. 8 ▸, the most negative ESP regions are concentrated around the carbonyl oxygen atoms. The strongest ESP minimum is −35.59 kcal mol^−1^, while the second carbonyl oxygen gives a local minimum of −30.63 kcal mol^−1^. These oxygen atoms are therefore the most probable acceptor sites for weak hydrogen-bonding inter­actions.

The positive ESP region is mainly located near the hydrogen atoms of the methyl­ene group situated between the two electron-withdrawing ester fragments, with an ESP maximum of 21.03 kcal mol^−1^. This polarization explains why this part of the mol­ecule may participate in weak C—H⋯O contacts in the solid state. Overall, the DFT results are consistent with the crystallographic analysis and show that the mol­ecular conformation, frontier orbitals and electrostatic potential are primarily governed by the benzoate ester fragment and the two carbonyl oxygen atoms.

## 6. Database survey

A search of the Cambridge Structure Database (CSD, version 6.01, November 2025, including February 2026 updates; Groom *et al.*, 2016 ▸) for structures containing the menthol-derived 2-isopropyl-5-methyl­cyclo­hexyl fragment revealed 1106 hits. The search was performed without refcode restrictions and additional filters. Among these entries, the crystal structure of menthol itself is deposited as BAVLOZ (Bombicz *et al.*, 1999 ▸). Several structurally related menthol-derived esters were also found, including AHILAF (Fuchs *et al.*, 2008 ▸), AJEXUJ (Dutkiewicz *et al.*, 2009 ▸) and QOVKAO (Xu *et al.*, 2009 ▸). These structures contain a menthyl cyclo­hexyl fragment attached to carbonyl-containing heterocyclic or aromatic fragments through an ester linkage. However, no analogous structure containing the menthyl —O—C(=O)—CH_2_—O—C(=O)—Ph fragment was found. Thus, the title compound represents a new menthol-based benzoate ester structure and provides an additional example for evaluating the influence of a bulky chiral menthyl group and a flexible ester–methyl­ene–ester linker on mol­ecular conformation and crystal packing.

## 7. Synthesis and crystallization

**Figure 9:**
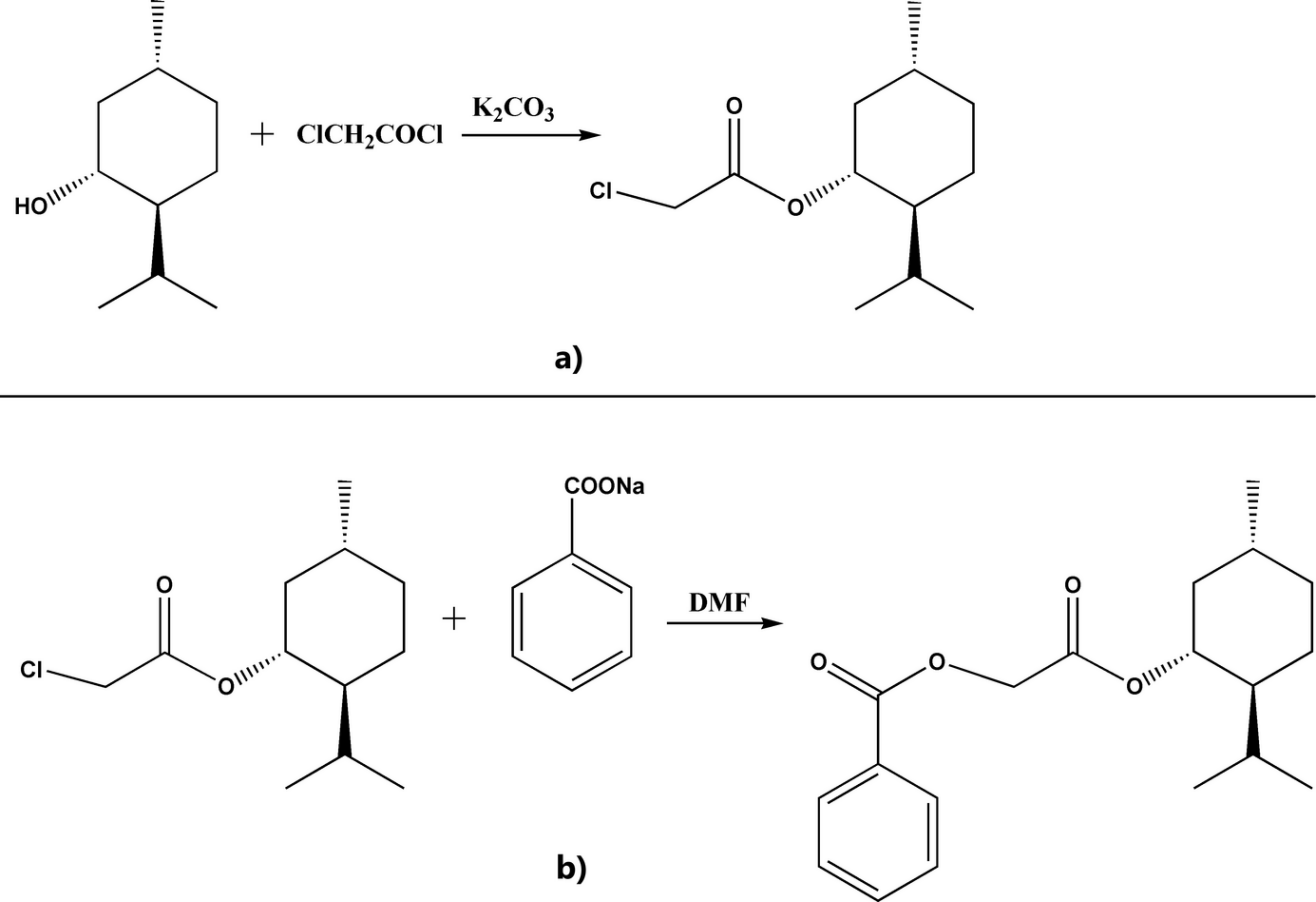
Synthesis scheme for (*a*) 2-(2-isopropyl-5-methyl­cyclo­hex­yloxy)-2-oxoethyl chloride from menthol and for (*b*) 2-(2-isopropyl-5-methyl­cyclo­hex­yloxy)-2-oxoethyl benzoate.

The title compound was synthesized in two steps. In the first step, menthol was converted into 2-(2-isopropyl-5-methyl­cyclo­hex­yloxy)-2-oxoethyl chloride (Fig. 9 ▸*a*). Menthol was dissolved in chloro­form and K_2_CO_3_ was added. Chloro­acetyl chloride was then added dropwise at 274–276 K, and the reaction mixture was subjected to ultrasonic irradiation for 2 h. The solvent was removed under reduced pressure, and the residue was recrystallized from methanol to give the inter­mediate in 86% yield. In the second step, the inter­mediate was refluxed with sodium benzoate in di­methyl­formamide for 11 h to afford the title compound (Fig. 9 ▸*b*). After filtration of the precipitated salt and evaporation of the solvent, the crude product was recrystallized from methanol to give the title compound in 82% yield. Colourless block-shaped crystals suitable for single-crystal X-ray diffraction analysis were obtained by slow evaporation of a methanol solution.

FT–IR spectroscopy confirmed the formation of the ester product. The broad O—H band of menthol at 3245–3600 cm^−1^ disappeared in the spectra of the synthesized esters. Menthyl chloro­acetate showed characteristic bands at 1745 cm^−1^ for the ester C=O group and 786 cm^−1^ for the C—Cl bond. In the title compound, the C—Cl band was absent, while strong absorptions at 1747–1717 cm^−1^ and 1207 cm^−1^ were observed for the ester C=O and C—O—C groups, respectively.

## 8. Refinement

Crystal data, data collection and structure refinement details are summarized in Table 2 ▸. No additional crystallographic symmetry was detected by *PLATON*/ADDSYM analysis (Spek, 2020 ▸), confirming the correctness of the refinement in *P*2_1_. Although using Cu radiation, the Flack parameter of 0.2 (4) is not sufficiently precise for an independent absolute-structure determination. Hence, the absolute configuration is assigned from the known configuration of the menthol precursor used in the synthesis. H atoms were placed in geometrically calculated positions and refined using a riding model, with C—H = 0.93–0.98 Å and *U*_iso_(H) = 1.2*U*_eq_(C) for aromatic, methyl­ene and methine H atoms, and 1.5*U*_eq_(C) for methyl H atoms.

## Supplementary Material

Crystal structure: contains datablock(s) I. DOI: 10.1107/S2056989026007528/wm5806sup1.cif

Structure factors: contains datablock(s) I. DOI: 10.1107/S2056989026007528/wm5806Isup2.hkl

Supporting information file. DOI: 10.1107/S2056989026007528/wm5806Isup3.cml

CCDC reference: 2535282

Additional supporting information:  crystallographic information; 3D view; checkCIF report

## Figures and Tables

**Table 1:** Hydrogen-bond geometry (Å, °)

*D*—H⋯*A*	*D*—H	H⋯*A*	*D*⋯*A*	*D*—H⋯*A*
C8—H8*A*⋯O3^i^	0.97	2.50	3.449 (8)	165

Symmetry code: (i) 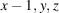.

**Table 2:** Experimental details

Crystal data	
Chemical formula	C_19_H_26_O_4_
*M*_r_	318.40
Crystal system, space group	Monoclinic, *P*2_1_
Temperature (K)	293
*a*, *b*, *c* (Å)	5.2145 (5), 14.9762 (13), 12.0221 (12)
β (°)	97.915 (9)
*V* (Å^3^)	929.91 (16)
*Z*	2
Radiation type	Cu *K*α
μ (mm^−1^)	0.63
Crystal size (mm)	0.30 × 0.22 × 0.16

Data collection	
Diffractometer	XtaLAB Synergy, Single source at home/near, HyPix3000
Absorption correction	Multi-scan (*CrysAlis PRO*; Rigaku OD, 2023 ▸)
*T*_min_, *T*_max_	0.577, 1.000
No. of measured, independent and observed [*I* > 2σ(*I*)] reflections	7401, 3369, 1523
*R*_int_	0.063
(sin θ/λ)_max_ (Å^−1^)	0.615

Refinement	
*R*[*F*^2^ > 2σ(*F*^2^)], *wR*(*F*^2^), *S*	0.059, 0.181, 0.88
No. of reflections	3369
No. of parameters	212
No. of restraints	1
H-atom treatment	H-atom parameters constrained
Δρ_max_, Δρ_min_ (e Å^−3^)	0.12, −0.13
Absolute structure	Flack *x* determined using 466 quotients [(*I*^+^)−(*I*^−^)]/[(*I*^+^)+(*I*^−^)] (Parsons *et al.*, 2013 ▸)
Absolute structure parameter	0.2 (4)

Computer programs: *CrysAlis PRO* (Rigaku OD, 2023 ▸), *SHELXT* (Sheldrick, 2015*a* ▸), *SHELXL* (Sheldrick, 2015*b* ▸), *OLEX2* (Dolomanov *et al.*, 2009 ▸) and *publCIF* (Westrip, 2010 ▸).

## supplementary crystallographic information

### 2-(2-Isopropyl-5-methylcyclohexyloxy)-2-oxoethyl benzoate Crystal data

**Table:**

C_19_H_26_O_4_	*F*(000) = 344
*M**_r_* = 318.40	*D*_x_ = 1.137 Mg m^−^^3^
Monoclinic, *P*2_1_	Cu *K*α radiation, λ = 1.54184 Å
*a* = 5.2145 (5) Å	Cell parameters from 1254 reflections
*b* = 14.9762 (13) Å	θ = 3.7–45.0°
*c* = 12.0221 (12) Å	µ = 0.63 mm^−^^1^
β = 97.915 (9)°	*T* = 293 K
*V* = 929.91 (16) Å^3^	Block, colourless
*Z* = 2	0.30 × 0.22 × 0.16 mm

### 2-(2-Isopropyl-5-methylcyclohexyloxy)-2-oxoethyl benzoate Data collection

**Table:**

XtaLAB Synergy, Single source at home/near, HyPix3000 diffractometer	3369 independent reflections
Radiation source: micro-focus sealed X-ray tube, PhotonJet (Cu) X-ray Source	1523 reflections with *I* > 2σ(*I*)
Mirror monochromator	*R*_int_ = 0.063
Detector resolution: 10.0000 pixels mm^-1^	θ_max_ = 71.6°, θ_min_ = 3.7°
ω scans	*h* = −5→6
Absorption correction: multi-scan (CrysAlisPro; Rigaku OD, 2023)	*k* = −18→18
*T*_min_ = 0.577, *T*_max_ = 1.000	*l* = −14→14
7401 measured reflections	

### 2-(2-Isopropyl-5-methylcyclohexyloxy)-2-oxoethyl benzoate Refinement

**Table:**

Refinement on *F*^2^	H-atom parameters constrained
Least-squares matrix: full	*w* = 1/[σ^2^(*F*_o_^2^) + (0.0867*P*)^2^] where *P* = (*F*_o_^2^ + 2*F*_c_^2^)/3
*R*[*F*^2^ > 2σ(*F*^2^)] = 0.059	(Δ/σ)_max_ < 0.001
*wR*(*F*^2^) = 0.181	Δρ_max_ = 0.12 e Å^−^^3^
*S* = 0.88	Δρ_min_ = −0.13 e Å^−^^3^
3369 reflections	Extinction correction: SHELXL2025/1 (Sheldrick, 2015)b, Fc^*^=kFc[1+0.001xFc^2^λ^3^/sin(2θ)]^-1/4^
212 parameters	Extinction coefficient: 0.0046 (17)
1 restraint	Absolute structure: Flack *x* determined using 466 quotients [(*I*^+^)-(*I*^-^)]/[(*I*^+^)+(*I*^-^)] (Parsons *et al.*, 2013)
Hydrogen site location: inferred from neighbouring sites	Absolute structure parameter: 0.2 (4)

### 2-(2-Isopropyl-5-methylcyclohexyloxy)-2-oxoethyl benzoate Special details

**Table:**

Geometry. All esds (except the esd in the dihedral angle between two l.s. planes) are estimated using the full covariance matrix. The cell esds are taken into account individually in the estimation of esds in distances, angles and torsion angles; correlations between esds in cell parameters are only used when they are defined by crystal symmetry. An approximate (isotropic) treatment of cell esds is used for estimating esds involving l.s. planes.

### 2-(2-Isopropyl-5-methylcyclohexyloxy)-2-oxoethyl benzoate Fractional atomic coordinates and isotropic or equivalent isotropic displacement parameters (Å^2^)

**Table:**

	*x*	*y*	*z*	*U*_iso_*/*U*_eq_	
O1	0.4222 (10)	0.5235 (3)	0.8708 (4)	0.1119 (16)	
O2	0.1142 (8)	0.6202 (3)	0.8031 (4)	0.0906 (13)	
O3	0.3660 (9)	0.5807 (3)	0.6239 (4)	0.0979 (14)	
O4	0.1136 (6)	0.4612 (2)	0.5820 (3)	0.0827 (12)	
C1	0.4340 (12)	0.6716 (4)	0.9457 (5)	0.0782 (16)	
C2	0.3136 (14)	0.7526 (4)	0.9474 (6)	0.095 (2)	
H2	0.157587	0.762651	0.901574	0.115*	
C3	0.4246 (18)	0.8198 (5)	1.0176 (8)	0.114 (2)	
H3	0.344587	0.875255	1.017734	0.137*	
C4	0.6493 (18)	0.8046 (6)	1.0860 (7)	0.114 (2)	
H4	0.721129	0.849777	1.133469	0.137*	
C5	0.7716 (17)	0.7244 (7)	1.0863 (8)	0.125 (3)	
H5	0.926514	0.714747	1.133082	0.150*	
C6	0.6621 (14)	0.6570 (5)	1.0157 (7)	0.106 (2)	
H6	0.743297	0.601716	1.015808	0.127*	
C7	0.3253 (13)	0.5965 (4)	0.8710 (6)	0.0861 (18)	
C8	0.0117 (11)	0.5516 (4)	0.7259 (6)	0.0903 (19)	
H8A	−0.157935	0.569278	0.689056	0.108*	
H8B	−0.007530	0.496697	0.766721	0.108*	
C9	0.1887 (11)	0.5361 (4)	0.6395 (5)	0.0755 (16)	
C10	0.2660 (9)	0.4341 (4)	0.4934 (6)	0.0749 (16)	
H10	0.446286	0.452788	0.514340	0.090*	
C11	0.1572 (12)	0.4782 (4)	0.3842 (6)	0.0881 (18)	
H11A	0.173263	0.542410	0.392606	0.106*	
H11B	−0.025558	0.464057	0.367451	0.106*	
C12	0.2928 (12)	0.4488 (5)	0.2864 (6)	0.0972 (19)	
H12	0.474460	0.467316	0.302069	0.117*	
C13	0.2850 (16)	0.3467 (5)	0.2806 (6)	0.109 (2)	
H13A	0.107070	0.327516	0.260043	0.131*	
H13B	0.382984	0.326842	0.222192	0.131*	
C14	0.3933 (12)	0.3036 (4)	0.3894 (6)	0.092 (2)	
H14A	0.575136	0.318881	0.406767	0.111*	
H14B	0.381061	0.239227	0.380975	0.111*	
C15	0.2534 (9)	0.3320 (3)	0.4868 (5)	0.0737 (16)	
H15	0.070648	0.316485	0.465378	0.088*	
C16	0.173 (2)	0.4932 (7)	0.1766 (8)	0.162 (4)	
H16A	0.274301	0.478556	0.118112	0.243*	
H16B	0.171433	0.556772	0.186515	0.243*	
H16C	−0.000620	0.472026	0.156354	0.243*	
C17	0.3404 (11)	0.2849 (4)	0.5982 (6)	0.0843 (18)	
H17	0.235801	0.309298	0.652834	0.101*	
C18	0.2859 (16)	0.1844 (4)	0.5908 (8)	0.124 (3)	
H18A	0.111647	0.174547	0.555433	0.186*	
H18B	0.307188	0.159319	0.665025	0.186*	
H18C	0.404571	0.156325	0.547392	0.186*	
C19	0.6234 (11)	0.3024 (6)	0.6447 (8)	0.132 (3)	
H19A	0.733311	0.274243	0.597302	0.198*	
H19B	0.659583	0.278477	0.719282	0.198*	
H19C	0.655074	0.365585	0.646728	0.198*	

### 2-(2-Isopropyl-5-methylcyclohexyloxy)-2-oxoethyl benzoate Atomic displacement parameters (Å^2^)

**Table:**

	*U*^11^	*U*^22^	*U*^33^	*U*^12^	*U*^13^	*U*^23^
O1	0.137 (4)	0.065 (3)	0.125 (4)	0.016 (3)	−0.014 (3)	−0.009 (3)
O2	0.085 (3)	0.075 (3)	0.109 (4)	0.008 (2)	0.001 (2)	−0.018 (3)
O3	0.100 (3)	0.076 (3)	0.121 (4)	−0.029 (2)	0.027 (3)	−0.013 (2)
O4	0.065 (2)	0.065 (2)	0.120 (3)	−0.0107 (18)	0.020 (2)	−0.020 (2)
C1	0.092 (4)	0.060 (3)	0.084 (4)	−0.005 (3)	0.015 (3)	−0.004 (3)
C2	0.117 (5)	0.074 (4)	0.094 (5)	0.004 (4)	0.009 (4)	−0.007 (4)
C3	0.149 (7)	0.070 (4)	0.122 (6)	−0.007 (5)	0.011 (6)	−0.020 (4)
C4	0.145 (7)	0.091 (6)	0.104 (6)	−0.031 (5)	0.007 (5)	−0.012 (5)
C5	0.123 (6)	0.119 (7)	0.125 (7)	−0.015 (6)	−0.014 (5)	−0.012 (6)
C6	0.107 (5)	0.094 (5)	0.110 (6)	0.005 (4)	−0.007 (5)	−0.012 (4)
C7	0.096 (4)	0.067 (4)	0.094 (5)	0.006 (3)	0.008 (4)	−0.005 (3)
C8	0.073 (3)	0.079 (4)	0.116 (5)	−0.005 (3)	0.006 (4)	−0.020 (4)
C9	0.067 (3)	0.056 (3)	0.104 (5)	−0.002 (3)	0.011 (3)	0.001 (3)
C10	0.054 (3)	0.065 (3)	0.107 (5)	−0.006 (3)	0.014 (3)	−0.013 (3)
C11	0.078 (3)	0.078 (4)	0.107 (5)	0.009 (3)	0.010 (3)	0.005 (4)
C12	0.095 (4)	0.095 (5)	0.100 (5)	0.011 (4)	0.008 (4)	0.000 (4)
C13	0.123 (5)	0.105 (6)	0.100 (6)	0.013 (4)	0.016 (4)	−0.017 (5)
C14	0.093 (4)	0.071 (4)	0.115 (6)	0.012 (3)	0.024 (4)	−0.005 (4)
C15	0.059 (3)	0.060 (3)	0.104 (5)	0.001 (3)	0.016 (3)	−0.010 (3)
C16	0.205 (9)	0.171 (9)	0.110 (7)	0.048 (8)	0.022 (6)	0.037 (7)
C17	0.070 (3)	0.077 (4)	0.109 (5)	0.002 (3)	0.022 (3)	0.002 (4)
C18	0.141 (6)	0.069 (4)	0.168 (8)	0.006 (4)	0.043 (6)	0.004 (5)
C19	0.076 (4)	0.164 (8)	0.153 (7)	0.003 (5)	0.002 (4)	0.043 (6)

### 2-(2-Isopropyl-5-methylcyclohexyloxy)-2-oxoethyl benzoate Geometric parameters (Å, º)

**Table:**

O1—C7	1.204 (7)	C11—H11B	0.9700
O2—C7	1.325 (8)	C11—C12	1.517 (9)
O2—C8	1.437 (7)	C12—H12	0.9800
O3—C9	1.177 (6)	C12—C13	1.531 (11)
O4—C9	1.347 (7)	C12—C16	1.532 (10)
O4—C10	1.470 (6)	C13—H13A	0.9700
C1—C2	1.367 (8)	C13—H13B	0.9700
C1—C6	1.377 (9)	C13—C14	1.498 (10)
C1—C7	1.502 (8)	C14—H14A	0.9700
C2—H2	0.9300	C14—H14B	0.9700
C2—C3	1.387 (10)	C14—C15	1.524 (8)
C3—H3	0.9300	C15—H15	0.9800
C3—C4	1.355 (11)	C15—C17	1.526 (8)
C4—H4	0.9300	C16—H16A	0.9600
C4—C5	1.360 (11)	C16—H16B	0.9600
C5—H5	0.9300	C16—H16C	0.9600
C5—C6	1.389 (11)	C17—H17	0.9800
C6—H6	0.9300	C17—C18	1.532 (9)
C8—H8A	0.9700	C17—C19	1.527 (8)
C8—H8B	0.9700	C18—H18A	0.9600
C8—C9	1.500 (9)	C18—H18B	0.9600
C10—H10	0.9800	C18—H18C	0.9600
C10—C11	1.509 (8)	C19—H19A	0.9600
C10—C15	1.533 (7)	C19—H19B	0.9600
C11—H11A	0.9700	C19—H19C	0.9600
			
C7—O2—C8	114.1 (5)	C11—C12—C16	111.4 (6)
C9—O4—C10	117.0 (4)	C13—C12—H12	108.1
C2—C1—C6	119.5 (6)	C13—C12—C16	112.9 (7)
C2—C1—C7	122.5 (6)	C16—C12—H12	108.1
C6—C1—C7	118.0 (6)	C12—C13—H13A	109.1
C1—C2—H2	120.1	C12—C13—H13B	109.1
C1—C2—C3	119.9 (7)	H13A—C13—H13B	107.8
C3—C2—H2	120.1	C14—C13—C12	112.7 (6)
C2—C3—H3	120.0	C14—C13—H13A	109.1
C4—C3—C2	120.1 (7)	C14—C13—H13B	109.1
C4—C3—H3	120.0	C13—C14—H14A	109.0
C3—C4—H4	119.5	C13—C14—H14B	109.0
C3—C4—C5	121.0 (8)	C13—C14—C15	112.8 (5)
C5—C4—H4	119.5	H14A—C14—H14B	107.8
C4—C5—H5	120.4	C15—C14—H14A	109.0
C4—C5—C6	119.1 (8)	C15—C14—H14B	109.0
C6—C5—H5	120.4	C10—C15—H15	106.4
C1—C6—C5	120.4 (8)	C14—C15—C10	107.3 (5)
C1—C6—H6	119.8	C14—C15—H15	106.4
C5—C6—H6	119.8	C14—C15—C17	115.5 (5)
O1—C7—O2	123.8 (6)	C17—C15—C10	114.2 (5)
O1—C7—C1	123.9 (6)	C17—C15—H15	106.4
O2—C7—C1	112.3 (5)	C12—C16—H16A	109.5
O2—C8—H8A	109.6	C12—C16—H16B	109.5
O2—C8—H8B	109.6	C12—C16—H16C	109.5
O2—C8—C9	110.4 (5)	H16A—C16—H16B	109.5
H8A—C8—H8B	108.1	H16A—C16—H16C	109.5
C9—C8—H8A	109.6	H16B—C16—H16C	109.5
C9—C8—H8B	109.6	C15—C17—H17	106.8
O3—C9—O4	124.8 (6)	C15—C17—C18	111.9 (6)
O3—C9—C8	126.4 (6)	C15—C17—C19	113.4 (5)
O4—C9—C8	108.8 (5)	C18—C17—H17	106.8
O4—C10—H10	109.3	C19—C17—H17	106.8
O4—C10—C11	109.5 (4)	C19—C17—C18	110.6 (6)
O4—C10—C15	106.8 (5)	C17—C18—H18A	109.5
C11—C10—H10	109.3	C17—C18—H18B	109.5
C11—C10—C15	112.5 (5)	C17—C18—H18C	109.5
C15—C10—H10	109.3	H18A—C18—H18B	109.5
C10—C11—H11A	109.0	H18A—C18—H18C	109.5
C10—C11—H11B	109.0	H18B—C18—H18C	109.5
C10—C11—C12	112.9 (5)	C17—C19—H19A	109.5
H11A—C11—H11B	107.8	C17—C19—H19B	109.5
C12—C11—H11A	109.0	C17—C19—H19C	109.5
C12—C11—H11B	109.0	H19A—C19—H19B	109.5
C11—C12—H12	108.1	H19A—C19—H19C	109.5
C11—C12—C13	108.2 (6)	H19B—C19—H19C	109.5
			
O2—C8—C9—O3	−10.7 (9)	C8—O2—C7—C1	176.8 (5)
O2—C8—C9—O4	168.7 (5)	C9—O4—C10—C11	−88.6 (5)
O4—C10—C11—C12	−176.2 (5)	C9—O4—C10—C15	149.3 (5)
O4—C10—C15—C14	175.8 (5)	C10—O4—C9—O3	−0.1 (8)
O4—C10—C15—C17	−54.9 (5)	C10—O4—C9—C8	−179.5 (5)
C1—C2—C3—C4	−1.3 (11)	C10—C11—C12—C13	54.1 (7)
C2—C1—C6—C5	−1.0 (11)	C10—C11—C12—C16	178.7 (7)
C2—C1—C7—O1	−177.2 (6)	C10—C15—C17—C18	171.5 (5)
C2—C1—C7—O2	4.4 (9)	C10—C15—C17—C19	−62.5 (7)
C2—C3—C4—C5	0.8 (13)	C11—C10—C15—C14	55.6 (6)
C3—C4—C5—C6	−0.5 (13)	C11—C10—C15—C17	−175.1 (4)
C4—C5—C6—C1	0.6 (13)	C11—C12—C13—C14	−54.2 (8)
C6—C1—C2—C3	1.4 (10)	C12—C13—C14—C15	57.9 (8)
C6—C1—C7—O1	2.0 (10)	C13—C14—C15—C10	−56.0 (7)
C6—C1—C7—O2	−176.4 (6)	C13—C14—C15—C17	175.4 (5)
C7—O2—C8—C9	−69.7 (7)	C14—C15—C17—C18	−63.5 (7)
C7—C1—C2—C3	−179.4 (6)	C14—C15—C17—C19	62.5 (7)
C7—C1—C6—C5	179.7 (7)	C15—C10—C11—C12	−57.6 (7)
C8—O2—C7—O1	−1.6 (9)	C16—C12—C13—C14	−177.9 (6)

### 2-(2-Isopropyl-5-methylcyclohexyloxy)-2-oxoethyl benzoate Hydrogen-bond geometry (Å, º)

**Table:**

*D*—H···*A*	*D*—H	H···*A*	*D*···*A*	*D*—H···*A*
C8—H8*A*···O3^i^	0.97	2.50	3.449 (8)	165

Symmetry code: (i) *x*−1, *y*, *z*.
